# Do Entrepreneurs’ Developmental Job Challenges Enhance Venture Performance in Emerging Industries? A Mediated Moderation Model of Entrepreneurial Action Learning and Entrepreneurial Experience

**DOI:** 10.3389/fpsyg.2019.01371

**Published:** 2019-06-11

**Authors:** Yanni Chen, Jianying Pan

**Affiliations:** ^1^School of Economics and Finance, Huaqiao University, Quanzhou, China; ^2^School of Management, Putian University, Putian, China

**Keywords:** developmental job challenges, entrepreneurial experience, entrepreneurial action learning, venture performance, mediated moderation model

## Abstract

Drawing on work experience theory and human capital theory, we developed a model of how developmental job challenges (DJC) enhance venture performance. Specifically, we developed and tested a mediated moderation model of DJC linking entrepreneurial action learning (EAL), entrepreneurial experience and venture performance. Based on a sample of Chinese entrepreneurs in emerging industries, we demonstrated that the developmental quality of entrepreneurial tasks had a positive relationship with venture performance and that this relationship was mediated by EAL. Furthermore, we found that entrepreneurs with prior entrepreneurial experience were more likely to engage in EAL behaviors and achieved better venture performance.

## Introduction

It is increasingly acknowledged that new ventures suffer a great deal in emerging industries (Dinlersoz and MacDonald, 2009; Forbes and Kirsch, 2011), as the context exposes them to high uncertainty, many challenges and few imitations (Forbes and Kirsch, 2011). Entrepreneurs must learn to quickly adapt to rapid changes, deal with diverse and major responsibilities, perform multiple roles, and resolve complex problems. It is well accepted that entrepreneurs learn from experience (Cope and Watts, 2000; Cope, 2011; Keith et al., 2016; Lafontaine and Shaw, 2016). Increasing research has proposed that entrepreneurial experience is important for the development of ventures (Ucbasaran et al., 2009; Cope, 2011). However, empirical research has given inconsistent results (Ucbasaran et al., 2009; Grégoire et al., 2010).

Prior research has enhanced our understanding of the importance of entrepreneurial experience, and there are some limitations. First, the majority of the studies have focused on the static stock of experience (Cope, 2005; Grégoire et al., 2010), neglecting the dynamic effects of job assignments. Second, researchers are mainly examining quantitative types of experience, such as the number of prior entrepreneurial experiences (Forbes, 2005) or the types of functional experiences (Dimov, 2009), which lead to inconsistent results. According to work experience theory (Tesluk and Jacobs, 1998), the quantitative component of work experience does not directly relate to complex (inconsistent) tasks. Third, the research so far has examined either the quantitative form of entrepreneurial experience (Ucbasaran et al., 2009) or the on-the-job experience (Keith et al., 2016) but not both in the same study. It is important to take an integrative perspective to better understand the mechanisms that underlie the relationship between entrepreneurial experience and venture performance.

Drawing on work experience theory (Tesluk and Jacobs, 1998) and human capital theory (Becker, 1964), we sought to propose and test a model of how developmental job challenges (DJC) affect the performance of new ventures in a sample of entrepreneurs from emerging industries in China. We conceptualized the entrepreneurs’ experience not only in quantitative terms, such as the number of start-ups they had begun or the number of years of functional job experience they had, but also in the developmental quality of the entrepreneurial tasks. We defined DJC as the extent to which start-up activities had developmental components that challenged and improved an entrepreneur’s skills and knowledge. We first developed and tested a mediation model linking DJC to entrepreneurial action learning (EAL) and venture performance. Then, we examined the moderating role of prior entrepreneurial experience in the mediation relationship.

The research makes three important contributions to extending a theory of entrepreneurial experience. First, the research extends existing understanding of entrepreneurial experience. We go beyond the traditional approaches of conceptualizing entrepreneur’s experience in terms of quantitative stock-like prior experience to the developmental quality of the entrepreneurial tasks. Second, the research advances our understanding of the impact of the interaction between quantitative and qualitative components of an entrepreneur’s experience. We did so by examining an integrated model of entrepreneurial experience by utilizing the work experience theory. The research not only examines the outcomes of the developmental quality of entrepreneurs’ on-the-job experience but also empirically analyzes the influence of its interaction with a quantitative component of entrepreneurial experience. Third, it extends research on DJC not only to entrepreneurs in the emerging industry but also to a non-Western cultural context, China. Given that the entry of new ventures is more common than that of mature firms in China’s emerging industries but with a higher failure rate (Dinlersoz and MacMillan, 2009; Forbes and Kirsch, 2011), it is important to help new ventures better perceive the developmental component of on-the-job experience and take more effective learning behaviors that lead to better venture performance.

## Literature Review and Hypothesis Development

### Work Experience Theory

Work experience is a critical variable in predicting work performance. Many studies have operationalized it in quantitative terms such as job tenure. Tesluk and Jacobs (1998) argued that this approach could not reflect the level of complexity and challenge encountered at work. In their research on the conceptualization of work experience, they identified three dimensions of the quantitative component (length of time or number of times that an assignment has been performed), qualitative component (perceptions of challenge and complexity), and interaction between qualitative and quantitative component (density and timing of an experience). Moreover, they provided a nomological net for work experience: contextual and individual factors are the antecedent variables; work motivation, knowledge and skills development, and work-related attitudes are immediate outcomes; work performance and career development are secondary outcomes.

### Developmental Job Challenge

A developmental job challenge, also referred as a challenging job experience, refers to one kind of on-the-job experience in which individuals are exposed to challenging jobs (McCauley et al., 1994; De Pater, 2009; Dragoni et al., 2009; Seibert et al., 2017). The term originates in the field of leadership development and is now popular in management and employee development (De Pater, 2009; Preenen et al., 2011; Carette et al., 2013). DJC shows individuals that there are gaps between expected states and present skills. To close the gaps, individuals are motivated to acquire new skills and knowledge and to adopt new approaches (Aryee and Chu, 2012). Thus, DJC refers to the developmental quality of a job as it provides opportunities for on-the-job learning (Dragoni et al., 2009).

The original research on DJC identified ten elements (McCauley et al., 1994). However, the majority of studies tend to use a condensed version with five elements: unfamiliar responsibilities, high levels of responsibility, creating changes, working across boundaries, and managing diversity (DeRue and Wellman, 2009; Dragoni et al., 2009; Cao and Hamori, 2016). Empirical findings have consistently linked DJC to many positive outcomes in different levels of the organization, such as supervisors’ leadership effectiveness (Seibert et al., 2017), employees’ task performance (Aryee and Chu, 2012), managers’ end-state competencies (Dragoni et al., 2009), and professionals’ organizational commitment (Cao and Hamori, 2016). While DJC boosts individual competencies and performance in an organizational context, whether DJC will enhance an entrepreneur’s performance needs more exploration.

### Entrepreneurial Action Learning in an Emerging Industry

EAL refers to how entrepreneurs learn from the problem solving process with the help of social interaction (Chen and Wang, 2015). While the definitions of entrepreneurial learning reflect a wide range of focuses (Wang and Chugh, 2014), EAL depicts learning behaviors in emerging industries with characteristics of high uncertainty, complexity, and unpredictability (Forbes and Kirsch, 2011). Although studies argue that entrepreneurial learning is a kind of learning-by-doing (Cope and Watts, 2000; Lafontaine and Shaw, 2016), some studies insist that entrepreneurs learn from social networks (Soetanto, 2017; Zozimo et al., 2017). EAL emphasizes that entrepreneurs not only learn through the actions toward challenges but also learn from interactions with other people, either through observation or through communication.

EAL is built upon the theory of action learning, which asserts that adults learn from solving complex problems with the help of learning sets (Pedler, 1991). Compared to traditional industries, emerging industries usually initiate new enterprises into a context characterized by higher uncertainty, fewer benchmarks, and more time pressure (Forbes and Kirsch, 2011). Under these conditions, entrepreneurs often face ill-structured problems that need to be responded to quickly. Entrepreneurs are so busy coping with difficulties that they have little time to join in formal learning. On-the-job learning comes to be their main learning source (Keith et al., 2016). Meanwhile, entrepreneurs have to interact with their context as they are located at the boundary between the internal venture and the external business networks. Through direct observation or communication with customers/suppliers/employees and other stakeholders, entrepreneurs can reframe the situation and develop better alternatives.

Chen and Wang (2015) have identified four elements of EAL: (a) information acquisition, in which entrepreneurs actively acquire new information, excellent practices and action-generated cues through various activities with the purpose of being able to better diagnose problems; (b) critical reflection, which refers to entrepreneurs recursively interpreting the collected information to infer the most plausible hypothesis; (c) systematic integration, which refers to the mental simulation in which entrepreneurs integrate the new hypothesis with reality, which generates new solutions to the problem; and (d) active verification, in which entrepreneurs make an effort to implement and verify the new solution with dedicated actions (Chen and Wang, 2015).

### Developmental Job Experience and Venture Performance

It is extensively supported in the literature that DJC is positively related to an individual’s performance (DeRue and Wellman, 2009; Dragoni et al., 2009; Carette et al., 2013). However, researchers thus far have not directly examined the relationship between developmental jobs and venture performance. New companies are generally small (Lans et al., 2008). Entrepreneurs are the main decision makers and managers, so they are important, as the venture needs them to recognize opportunities, assemble resources and exploit opportunities (Frese and Gielnik, 2014). According to upper echelon theory (Hambrick and Mason, 1984; Hambrick, 2007), we suggest that venture performance can be represented by an entrepreneur’s performance.

The following two reasons describe why challenge assignments are believed to augment venture performance. First, DJC forces entrepreneurs to increase their effort level to match the demanding tasks, which will result in better venture performance. Entrepreneurs’ actions are goal-oriented behaviors (Frese, 2011). Challenging job experiences indicate that entrepreneurs have perceived the difficulties they face in achieving their goals. According to goal-setting theory, difficult goals force individuals to focus on the selected task, exert a higher level of effort, persist in achieving the selected goal and develop new strategies, which will lead to better venture performance (Locke, 1968).

Second, according to work experience theory (Tesluk and Jacobs, 1998), the developmental dimension of qualitative experience will lead to work performance (Aryee and Chu, 2012). Oftentimes, entrepreneurs have to play different roles that have unfamiliar responsibilities (Zahra et al., 2018). Entrepreneurs have to face novel and complex assignments that might force them to consider the pros and cons of different options, to try new ways of acting and to reflect on feedback. These activities provide them with more opportunities to acquire new knowledge, practice skills and augment competencies (Dragoni et al., 2009). Therefore, compared to consistent tasks, these activities are more strongly related to performance on more inconsistent tasks (Tesluk and Jacobs, 1998; Aryee and Chu, 2012). Therefore, we propose the following:

*Hypothesis 1.* Developmental job challenge is positively related to venture performance.

### The Mediation Effect of Entrepreneurial Action Learning

DJC represents the learning opportunities in the areas of management and leadership development. Many empirical studies have consistently shown that DJC facilitates on-the-job learning (McCauley et al., 1994; DeRue and Wellman, 2009; Preenen et al., 2011). This is because such experience forces individuals to step out of their comfort zones to develop new ways of coping with problems (Dragoni et al., 2009). Moreover, this kind of experience also provides them with abundant learning resources, such as experimentation opportunities and diverse feedback.

According to work experience theory, learning behaviors are the direct outcome of work experience (Tesluk and Jacobs, 1998). Therefore, we suggest that DJC facilitates EAL. It is increasingly acknowledged that entrepreneurs learn from experience (Keith et al., 2016). However, two entrepreneurs may not learn equally from carrying out a similar job, as they may be presented with different challenging opportunities. In developmental jobs, one entrepreneur may learn more, as he/she has a greater “density” of experiences (Tesluk and Jacobs, 1998). The more entrepreneurs perceive the challenges they face, the more they will learn to reduce them, as any challenge may influence a venture’s survival. We suggest that this relationship will be significant in an emerging industry because the learning environment of an emerging industry is very dynamic, complex and uncertain. Empirical studies show that in traditional industries characterized by stability and redundancies, entrepreneurs’ ability to acquire new information and make creative ideas declines as their entrepreneurial experiences increase (Ucbasaran et al., 2009).

EAL benefits a venture’s growth because it helps an entrepreneur acquire knowledge and opportunities. According to work experience theory, learning is the immediate outcome of on-the-job experience, and work performance is the secondary outcome (Tesluk and Jacobs, 1998). This is consistent with human capital theory, in which skills and knowledge from learning is closely related to job performance (Becker, 1964). EAL refers to how entrepreneurs learn through problem solving in an emerging industry. With little time and energy, EAL represents the main forms of on-the-job learning behavior. EAL helps entrepreneurs to accumulate task-related human capital, which has a positive effect on success (Unger et al., 2011). Therefore, we postulate our second hypothesis.

*Hypothesis 2.* Entrepreneurial action learning mediates the relationship between developmental job challenges and venture performance.

### Entrepreneurial Experience’s Moderating Effect

What knowledge and experience entrepreneurs have will impact the process of opportunity recognition and exploitation (Shane and Venkatarman, 2000). Therefore, we incorporate entrepreneurial experience into our model. According to human capital theory, prior experience as an entrepreneur is an important domain-specific knowledge (Hmieleski et al., 2015). Such experience helps entrepreneurs be more familiar with the “liability of newness” and affects how they interpret and resolve problems (Forbes, 2005). A case study shows that entrepreneurs with prior entrepreneurship experience will be more product oriented, have better financing skills and be more balanced in essential business skills (Lamont, 1972). Therefore, it is important to study the impact of entrepreneurial experience.

However, the empirical findings have been inconsistent, as some studies have supported the expected relationship (Grégoire et al., 2010), while others have shown that prior experience could be negatively associated with entrepreneurial success (Rerup, 2005; Hmieleski et al., 2015). According to work experience theory, Tesluk and Jacobs (1998) propose that the interaction of the quantitative components and qualitative components of work experience are likely to impact subsequent outcomes, such as learning. Therefore, we propose that prior entrepreneurial experience, as a quantitative component of work experience, has a buffering effect on the relationship between DJC and EAL.

Previous research indicates that entrepreneurs have to make fast decisions and take quick actions in highly uncertain context (Mcmullen and Shepherd, 2006). In fact, top managers utilize more information and develop more alternatives in the high-velocity environment than the ones in a stable environment (Eisenhardt, 1989). Following this logic, on-the-job experiences abundant with DJC would place entrepreneurs at a high risk for cognitive overload in emerging industries, not only because such experiences induced off-task anxieties but because they require entrepreneurs to take judgmental decisions and respond to changing environment simultaneously. Entrepreneurs with prior entrepreneurial experience will demonstrate higher tolerance for the uncertainty and risks that come from challenges (Dimov, 2009). When faced with challenging situations, they would respond with more cautious behaviors that contribute to finding more information and deriving more insights. Indeed, entrepreneurs with prior entrepreneurial experience will be more effective in searching information and identifying opportunities than novice entrepreneurs (Ucbasaran et al., 2003). In contrast, novice entrepreneurs would be more likely to view challenging situations as risky and overwhelming and thus easily divert their attention away from the task. They worry about possible failures and evaluation anxieties, rather than address to the challenges and learn from the process. For these reasons, we hypothesized as follows:

*Hypothesis 3.* Entrepreneurial experience will strengthen the relationship between developmental job challenges and entrepreneurial action learning.

Again, using the theory of work experience, we propose that entrepreneurial experience moderates the relationship between DJC and venture performance. This is because work performance is the indirect outcome of the interaction between quantitative and qualitative components of work experience. Building on the above analysis, we hypothesize the following:

*Hypothesis 4.* Entrepreneurial experience will strengthen the relationship between developmental job challenges and venture performance.

Combining Hypotheses 3 and 4 together, we suggest that the effect of moderation is mediated by EAL. This is because on-the-job learning such as EAL is the immediate outcome of work experience (Tesluk and Jacobs, 1998). This implies that entrepreneurial experience has an overall moderation effect on venture performance, produced by the mediating process. When we control for the mediation effect of EAL, the residual moderation of the entrepreneurial experience’s effect on the dependent variable is reduced compared to the moderation effect for the original main effect. This means that the moderation effect of entrepreneurial experience is mediated by the EAL. This implies that there is a mediated moderation effect (Muller et al., 2005). Based on our analysis, we propose a mediated moderation model containing both moderation and mediation effects.

*Hypothesis 5.* Entrepreneurial action learning mediates the moderating effect of entrepreneurial experience on the relationship between developmental job challenges and venture performance.

**FIGURE 1:**
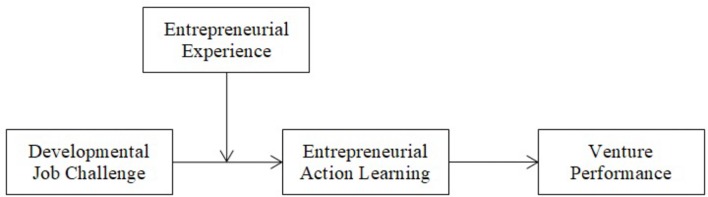
The hypothesized model.

Figure 1 shows the complete model of this research.

## Materials and Methods

### Data Collection and Sample Demographics

We selected entrepreneurs from emerging industries in China as our sample. Their industries had to meet the standards of China’s strategic emerging industries policy.^1^ The age of the respondents’ ventures had to be less than 8 years. This time period was chosen because previous research indicated that new ventures would become a mature business within the first 8 years (Chrisman et al., 1998). This study was carried out in accordance with the recommendations of “Ethic of guidelines, The Institutional Review Board of the Department of E-Commerce at Huaqiao University” with written informed consent from all participants. The Institutional Review Board of the Department of E-Commerce at Huaqiao University approved the protocol for this study.

We mainly collected data from the city of Hangzhou, which is named by “Chinese Silicon Valley.”^2^ Because respondents were very busy and hard to find, we used convenience sampling. We chose opportunities where entrepreneurs were already getting together, such as at emerging industrial parks, venture capital Roadshow competitions, training programs for emerging industrial entrepreneurs and so on. Specifically, we collected data mainly from 12 parks, two VC roadshows and two training programs. After getting permissions from the park’s management committee, we knocked at the door of every company there. On average, there were nearly 80 new ventures in each park, 25 entrepreneurs were in the office when we got there, and 14 of them were available and accepted the survey. When we surveyed from training programs and VC roadshows, almost all entrepreneurs accepted the survey.

For every respondent, we first asked her/him whether s/he was an entrepreneur. If s/he was, we introduced ourselves and described the purpose of the survey. After the respondents agreed to participate, we informed them of the rules and notices in filling out the forms. We also provided each of them a gift to represent our gratitude. Each questionnaire was completed within 1 h. As venture performance was an important variable in our model, we asked the respondent to report his/her role in the questionnaire. There were three options: individual entrepreneur, team leader of entrepreneurial team and team member of entrepreneurial team. If s/he chose the third option, the following sentence indicated that s/he should terminate the survey.

We gathered data from 230 ventures: 163 of them came from parks, 14 from roadshows and 43 from training programs.^3^ We eliminated surveys that had large portions of missing data and limited samples to emerging industries, resulting in 197 entrepreneurs. The final sample consisted of 51.30% male entrepreneurs. 33.0% of them were individual entrepreneurs. All respondents were, on average, 30.16 years old, and 59.5% had obtained a bachelor’s degree. The average age of the ventures was 29.96 months old, 44.20% ventures’ revenues were between RMB 30 million yuan and RMB 100 million yuan, and 70.10% ventures had fewer than 10 employees. In addition, 35.0% ventures belonged to digital innovation industry, 23.4% belonged to new generation information technology industry, 18.8% belonged to new energy industry and new material industry and 16.2% belonged to related services industry.

### Measures

We used a Chinese language version of the questionnaire. All original English versions of the items followed the translation-back-translation procedure.

#### Developmental Job Challenges

This variable was measured by seven items developed by McCauley et al. (1994). Two example items are “You have to manage something with which you are unfamiliar” and “You have to lay off a significant number of your people.” A 5-point Likert scale was used to measure the items, ranging from 1 (*very little*) to 5 (*a great deal*). The Cronbach’s alpha was 0.82.

#### Entrepreneurial Action Learning

The EAL scale used in this research was originally in Chinese. Fourteen items measured the four elements of information acquisition (e.g., “I frequently interact with others to acquire entrepreneurial information”), critical reflection (e.g., “I often adopt advice from others to rethink the problems that I have met”), systematic integration (e.g., “I adjust my plan systematically when I get new information”) and active verification (e.g., “I overcome difficulties to get my entrepreneurial ideas realized the best way possible”). We averaged the fourteen items to form a single score. Each entrepreneur indicated the frequency of each of these behaviors on a 5-point Likert scale ranging from 1 (*strongly disagree*) to 5 (*strongly agree*). The reliability was 0.85.

#### Venture Performance

All of our respondents were from private firms, and there was little or no published financial data. Moreover, entrepreneurs are reluctant to share their objective data (Smart and Conant, 1994). So we have to use subjective measure to asses venture performance. Although Reiter-Palmon et al. (2012) argue that whether self-perception of creative/work performance is correlated with objective measures depends on two assumptions. One is that the respondents are aware of questions. The other is that the respondents are willing to report them accurately. Our research met these two assumptions. First, when crafting the questionnaire, we have conducted a pretest with five entrepreneurs and interviewed them after that. We found that they comprehend the measurement correctly. Second, we guaranteed the confidentiality and anonymity of the survey. We believed our respondents would be willing to report them accurately.

Recognizing that new ventures failed a lot in emerging industries, we assessed financial performance by asking respondents to rate profitability growth, sales growth and market share growth on a 5-point Likert scale from 1 = *not at all* to 5 = *very much* (Poon et al., 2006; Keh et al., 2007). Respondents reported the data by benchmarking their own venture performance to their major competitors. Although there are limitations to the subjective, self-report measurement method, earlier research shows that top manager’s perceptions of the performance are highly consistent with objective measurement (Wall et al., 2004). The Cronbach’s alpha was 0.79.

#### Prior Entrepreneurial Experience

There are two methods of measuring entrepreneurial experience: (1) the number of new ventures founded previously, and (2) whether they have ever started a venture, indicated by a dummy variable. Both of them were widely used in previous research (Davidsson and Honig, 2003; Hmieleski and Baron, 2009; Farmer et al., 2011). However, whether prior entrepreneurial experience would impact the learning behaviors or venture performance depends on the external environment conditions (Ucbasaran et al., 2003). Experienced/habitual entrepreneurs would be quite dangerous when they transfer past experience to a different context or when the environment changes (Dawes, 1988). As our research focused on emerging industries characterized by higher uncertainty, fewer benchmarks, and more time pressure (Forbes and Kirsch, 2011), we took the second approach because the task-specific human capital provided by the first new venture experience had a greater impact than each subsequent experience (Brüderl et al., 1992). Following Forbes (2005), entrepreneurs with prior startup experience were allocated a value of “1,” while entrepreneurs with no such experience were allocated a value of “0.”

#### Control Variable

To examine each entrepreneur’s unique contribution in the model, we controlled for the effects of the gender (“male” = 0, “female” = 1), age (in years) and educational attainment (1 = “middle school,” 2 = “high school,” 3 = “associate’s degree,” 4 = “bachelor’s degree,” 5 = “master’s degree or above”) of each respondent. Additionally, because entrepreneurs who have a parent with entrepreneurial experience might have special business experience that influences their learning behaviors and performance (Kim et al., 2006), we dummy-coded parent entrepreneurship as 1 when a respondent had one parent as an entrepreneur and 0 when they did not. We also controlled for firm age as the number of months between the time when the venture was started and the time of our survey.

### Common Method Variance

Despite the variables we measured are not personally sensitive questions, and the variables are relevant to job characteristics which are partially independent of method effects (Glick et al., 1986), the method we used might lead to common method bias. According to Podsakoff et al. (2003), we adopted three procedural remedies to reduce the plausibility of the method bias.

First, a pretest was conducted to reduce item ambiguity by asking five entrepreneurs to fill out the questionnaire. They were interviewed afterward for feedback regarding the items and the questionnaire as a whole. The questionnaire was then modified accordingly to ensure item clarity. Second, we used different scale anchors in different variables’ measurement to avoid the respondents’ mapping judgements. Third, when we informed the rules to the respondents, we guaranteed the confidentiality and anonymity of the survey. And also, we emphasized that there were no right or wrong answers.

As procedural remedies are impossible to eliminate all forms of common variance bias, we also took statistical remedies to control for CMV. We conducted a one-factor model (see Harman, 1967) to make sure that common method bias would not nullify our findings. The poor results (χ^2^ = 1215.25, CFI = 0.47, TLI = 0.38, RMSEA = 0.14, and SRMR = 0.15) indicated that no single factor can explain a majority of the variance.

## Results

### Descriptive Statistics

Table 1 presents the means, standard deviations, and bivariate correlations among the variables. DJC was significantly related to EAL (*r* = 0.54, *p* < 0.001) and venture performance (*r* = 0.43, *p* < 0.001), suggesting that DJC facilitated the learning behaviors and performance. The linkage between EAL and venture performance was significant (*r* = 0.47, *p* < 0.001). Entrepreneurial experience was not significantly correlated with DJC and venture performance. According to Hayes (2013), a moderator does not necessarily correlate with independent and mediating variables.

**TABLE 1:** Descriptive statistics and correlation.^a^

**Variables^b^**	**M**	**SD**	**1**	**2**	**3**	**4**	**5**	**6**	**7**	**8**
1. Gender	0.52	0.50								
2. Age of entrepreneur	30.16	5.15	0.07							
3. Parent entrepreneurship	0.44	0.50	–0.01	–0.12						
4. Education	3.68	0.68	0.08	–0.01	–0.00					
5. Firm age (months)	29.96	16.02	0.12	0.35^∗∗∗^	–0.13	–0.08				
6. Developmental job challenges	3.77	0.60	–0.07	–0.03	–0.07	–0.09	–0.05			
7. Entrepreneurial experience	0.45	0.50	0.05	0.15^*^	–0.06	0.06	0.05	−0.00		
8. Entrepreneurial action learning	3.81	0.48	–0.07	0.07	–0.06	–0.11	–0.00	0.54^∗∗∗^	0.08	
9. Venture performance	3.45	0.61	–0.10	–0.08	–0.02	–0.07	–0.03	0.43^∗∗∗^	0.15^*^	0.47^∗∗∗^

^a^*n* = 197. ^b^For gender, 0 = male, 1 = female; parent entrepreneurship, 1 = at least one parent has entrepreneurial experience, 0 = both parents have no entrepreneurial experience. ^*^p < 0.05; ^∗∗∗^*p* < 0.001; two-tailed tests.

### Hypotheses Testing

We conducted a multivariate multiple regression technique to analyze Hypothesis 1. Table 2 presents the estimates of the relationship between DJC and venture performance. Model 4 comprised five control variables. We added DJC in Model 5; this addition provided a significantly better fit over Model 4. The result indicated that DJC was significantly related to venture performance (β = 0.41, *p* < 0.001). Therefore, the findings offer support for Hypothesis 1.

**TABLE 2:** Hierarchical multiple regression analysis.^a^

	**Entrepreneurial Action Learning**			**Venture Performance**				
	**Model 1**	**Model 2**	**Model 3**	**Model 4**	**Model 5**	**Model 6**	**Model 7**	**Model 8**
**Variables**								
**Step 1 (controls)**								
Gender	–0.09	–0.05	–0.04	–0.06	–0.05	–0.07	–0.05	–0.10
Age of entrepreneur	0.09	0.10	0.09	–0.10	–0.07	–0.06	–0.08	–0.05
PE	–0.08	–0.03	–0.02	–0.04	0.00	0.01	0.01	0.02
Education	–0.11	–0.06	–0.07	–0.05	–0.02	–0.04	0.03	–0.02
Firm age	–0.05	0.01	–0.01	–0.02	0.00	–0.01	–0.02	0.01
**Step 2 (main effect)**								
DJC		0.52^∗∗∗^	0.36^∗∗∗^		0.41^∗∗∗^	0.23^*^	0.24^∗∗∗^	0.13
**Step 3 (moderator)**								
EE			0.05			0.15^*^		0.14^*^
**Step 4 (interaction)**								
DJC × EE			0.20^*^			0.23^*^		0.17
**Step 5 (mediator)**								
EAL							0.32^∗∗∗^	0.29^∗∗∗^
*R*^2^	0.03	0.29	0.31	0.02	0.18	0.22	0.25	0.28
Adjusted *R*^2^	0.01	0.27	0.28	–0.01	0.15	0.19	0.22	0.25
*F*	1.33	12.97^∗∗∗^	10.50^∗∗∗^	0.75	6.84^∗∗∗^	6.61^∗∗∗^	8.95^∗∗∗^	7.98^∗∗∗^
Δ*R*^2^	–	0.26	0.02	–	0.16	0.04	0.07^b^	0.06^c^
Δ*F*	–	68.75^∗∗∗^	2.49	–	36.58^∗∗∗^	5.04^∗∗^	17.95^∗∗∗^	14.93^∗∗∗^

^a^*n* = 197. ^b^Compared with Model 5. ^c^Compared with Model 6. PE = parent entrepreneurship; DJC = developmental job challenges; EE = entrepreneurial experience; EAL = entrepreneurial action learning. ^*^p < 0.05; ^∗∗∗^*p* < 0.001; two-tailed tests.

To test the mediation effect, we followed the steps proposed by Baron and Kenny (1986). As shown in Model 2, DJC was significantly related to EAL (β = 0.52, *p* < 0.001). After controlling for the effect of DJC, EAL was significantly related to venture performance (β = 0.32, *p* < 0.001; Model 7). Meanwhile, the effect of DJC on venture performance decreased to (β = 0.24, *p* < 0.001; Model 7), indicating partial mediation. As our sample size was small, we also used Preacher and Hayes’s (2008) SPSS macro PROCESS to test the indirect effect. We conducted a bootstrapping method to respond to the normal distribution hypothesis. As presented in Table 3, the results showed that there was a significant indirect effect via EAL with 95% bias-corrected confidence intervals [0.10, 0.24] based on 2,000 bootstrapped samples. The outcomes supported Hypothesis 2.

**TABLE 3:** Unstandardized bootstrapping estimates for moderation, mediation, and mediated moderation.^a^

**Moderation**								
	**Test of interaction term**			**Conditional effect at the levels of EE**				
		**95% Bias-corrected CI**					**95% Bias-corrected CI**	
**Paths**	**Effect (ΔF)**	**LLCI**	**ULCI**	**EE level**	**Effect**	**SE**	**LLCI**	**ULCI**
DJC × EE → VP	0.29(4.86^∗∗^)	0.03	0.55	0	0.23	0.10	0.03	0.43
				1	0.52	0.08	0.35	0.68
DJC × EE → EAL	0.21(4.34^*^)	0.01	0.40	0	0.28	0.08	0.13	0.43
				1	0.49	0.06	0.36	0.61

**Mediation**								
			**95% Bias-corrected CI**		**Normal theory test for indirect effect**			
**Path**	**Indirect effect**	**SE**	**LLCI**	**ULCI**	**Effect**	**SE**	**z**	***p***

DJC → EAL → VP	0.16	0.04	0.10	0.24	0.16	0.04	3.75	0.000

**Mediated moderation**								
	**Index of moderated mediation (mediated moderation)**			**Conditional indirect effect at the level of EE**				
		**95% Bias-corrected CI**					**95% Bias-corrected CI**	
**Path**	**Index(SE)**	**LLCI**	**ULCI**	**EE level**	**Effect**	**SE**	**LLCI**	**ULCI**

DJC × EE → EAL → VP	0.08(0.04)	0.02	0.17	0	0.11	0.03	0.06	0.18
				1	0.19	0.04	0.12	0.29

^a^*n* = 197. PE = parent entrepreneurship; DJC = developmental job challenges; EE = entrepreneurial experience; EAL = entrepreneurial action learning; VP = venture performance; LLCI and ULCI = lower and upper levels of confidence intervals, respectively. ^*^p < 0.05; ^∗∗^*p* < 0.01.

**FIGURE 2:**
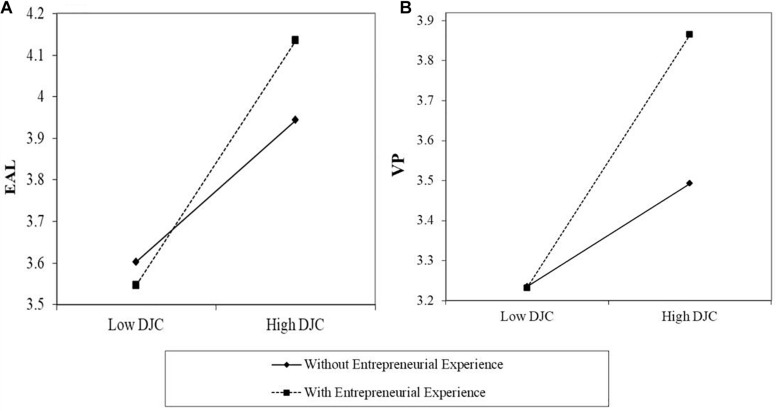
**(A)** The moderation effect of entrepreneurial experience (Moderator) on the relationship between DJC [developmental job challenges (IV)] and EAL [entrepreneurial action learning (DV)]. **(B)** The moderation effect of entrepreneurial experience (Moderator) on the relationship between DJC [developmental job challenges (IV)] and venture performance (DV).

For Hypotheses 3 and 4, we tested the moderating role of entrepreneurial experience. The data (see Model 3 and Model 6 in Table 2) revealed that entrepreneurial experience had a significant moderating effect on the relationship between DJC and EAL (β = 0.20, *p* < 0.05) and on the relationship between DJC and venture performance (β = 0.23, *p* < 0.05). As hypothesized, the effect of DJC was greater for entrepreneurs with entrepreneurial experience (see Figures 2A,B), as indicated by the steeper slopes for these entrepreneurs than for new entrepreneurs. Both moderation effects were confirmed by bootstrapping tests with 95% BCCI of [0.03, 0.55] and [0.01, 0.40]. Therefore, the results support Hypotheses 3 and 4.

We proposed that the overall effect of DJC on venture performance would be moderated by entrepreneurial experience and that this interaction would be due to the effect of the mediator (EAL) and the moderation of the mediator effect on the EAL by entrepreneurial experience. According to Muller et al. (2005), we confirmed that this was a mediated moderation model.

We still used the SPSS macro PROCESS developed by Preacher and Hayes (2008) to test the mediated moderation effect. The macro allows us to test the whole model at once. As shown for DJC in Models 3 and 8 in Table 2, significant effects were found for EAL by entrepreneurial experience (as previously confirmed); in addition, the values for EAL remained significant in Model 8 (β = 0.29, *p* < 0.001). According to Muller et al. (2005), the data meet the conditions for a mediated moderation effect. Therefore, the moderation effect of entrepreneurial experience on the relationship between DJC and EAL (β = 0.20, *p* < 0.05) became nonsignificant (β = 0.17, *p* > 0.05), demonstrating fully mediated moderation. Combining these results, we concluded that there were fully mediated moderation effects for DJC. Thus, Hypothesis 5 was supported.

## Discussion

Drawing on work experience theory and human capital theory, we proposed and tested a model to examine the relationship between DJC, entrepreneurial experience, EAL and venture performance in a sample of entrepreneurs from emerging industries in China. The results demonstrated support for the hypothesized mediated moderation model. Specifically, DJC was positively related to venture performance, and EAL partly mediated the relationship. In turn, prior entrepreneurial experience moderated the relationship between DJC and venture performance. Furthermore, the moderation effect was fully mediated by EAL.

### Theoretical Implications

Our research provides three theoretical contributions to the literature on DJC, entrepreneurial experience and EAL. First, our research advances current entrepreneurial experience research by going beyond the traditional approaches of conceptualizing entrepreneur’s experience in terms of quantitative stock-like prior experience to the developmental quality of the entrepreneurial tasks. By utilizing work experience theory, we examined the impact of the quality of an entrepreneur’s on-the-job experience on venture performance. The results show that venture performance could be enhanced if entrepreneurial tasks are rich in developmental dimensions. The finding provides a good empirical basis for future research on how the qualitative component of on-the-job experience facilitates the development of entrepreneurs and ventures. Meanwhile, the research opens the door for more considerate attention to the other type of prior experience in relation to developmental assignment quality.

Second, this research advances our understanding of the impact of the interaction between quantitative and qualitative components of an entrepreneur’s experience. Although an entrepreneur’s experience plays an important role in entrepreneurial process (Cope, 2005; Pittaway and Thorpe, 2012), there is no well-developed and empirically supported theory on how entrepreneurial experience develops ventures. We found that entrepreneurs with prior entrepreneurial experience would be more likely to perform EAL behaviors when they perceived challenges from the assignments. Moreover, we found that the relationship between DJC and venture performance was stronger if the entrepreneur had prior entrepreneurial experience. The findings provide empirical evidence that both entrepreneurial characteristics and entrepreneurial tasks are important for understanding how entrepreneurs learn and develop.

Third, the study contributes to the theory development of EAL in China’s emerging industries. We extend prior conceptual work on entrepreneurial learning by empirically demonstrating DJC as the antecedent and venture performance as the outcome. Specifically, we found that the challenges from an assignment directly impacted the learning behaviors and that prior entrepreneurial experience plays a moderating role in this relationship. This is partly consistent with the proposition from prior research that entrepreneurs learn from experience. Therefore, we empirically differentiate the distinct impact of entrepreneurial learning from different components of entrepreneurial experience.

### Practical Implications

This research explores the mechanisms that underlie the relationship between the developmental quality of on-the-job experience and venture performance in a sample of Chinese entrepreneurs in the emerging industry. It provides empirical evidence that challenging assignments can have positive impacts, especially in terms of increasing EAL behaviors and enhancing venture performance.

Specifically, the research provides guidance for entrepreneurs to improve venture performance through entrepreneurial assignments. Our results suggest that there is a need for entrepreneurs to actively seek DJC for developing ventures. Performing such assignments can motivate them to learn more from the on-the-job experience and benefit the venture. Moreover, given that EAL partly mediates the relationship between DJC and venture performance and fully mediates the moderation effect of prior entrepreneurial experience, we suggest that entrepreneurs adopt EAL behaviors when they are exposed to challenging assignments. Finally, our research provides implications for entrepreneurs with prior entrepreneurial experience. Since the results show that on-the-job experience is more important than static stock-like prior experience, entrepreneurs should focus more on experience from current assignments.

### Limitations and Future Research

Although the research arrives at interesting findings, there are inevitably some limitations associated with it. While we are discussing the limitations, we will provide future directions.

First, we used cross-sectional self-report method research design which might lead to common method variance (Podsakoff et al., 2003). Despite it is still the most valid and useful method in accessing individual perceptions (Spector, 1994), and we have taken procedural and statistical remedies to control for the common method bias, we cannot eliminate all forms of method bias. Future research should consider the use of more objective measures.

Second, since this is a cross-sectional study, the uncertainty of causal relationships exists. It may be possible that entrepreneurs who actively engaged in EAL behaviors will perceive more challenges from the assignments rather than the developmental challenges driving them to learn. We could not deny this possibility, but the research findings are consistent with the theory logic proposed by work experience theory. Future research should design longitudinal studies to clarify these causal relationships.

Third, our conclusions are limited to Chinese entrepreneurs in emerging industries. The cultural context and market environments are different from those in a Western context. We are not sure whether the model will be generalizable to other countries. Future research can replicate the model by utilizing a cross-national sample. Third, since the entrepreneurs’ experiences and learning behaviors are contextualized, we did not incorporate contextual variables into our model. Future research should examine environmental conditions such as environmental dynamics.

## Author Contributions

All authors listed have made a substantial, direct and intellectual contribution to the work, and approved it for publication.

## Conflict of Interest Statement

The authors declare that the research was conducted in the absence of any commercial or financial relationships that could be construed as a potential conflict of interest.

## Footnotes

1 In order to structure the economy and develop the promising industries, China has released the catalogs of strategic emerging industries (SEIs) in her Five-Year Development Plan. The 13th Plan in 2016 released the SEI catalog with nine sectors.

2 There are nearly 20 big industrial/science parks and many small start-up incubators in this city. VC roadshows are very hot in this city. And the government there provides many free training programs for young entrepreneurs with good potential.

3 There are nearly 30 entrepreneurs in each training program. All of them are selected from entrepreneur alliance of Zhejiang province. This alliance has more than 5,000 entrepreneurs.
